# The effect of low- dose tranexamic acid on postoperative blood loss in patients treated with clopidogrel and aspirin

**DOI:** 10.22088/cjim.10.2.156

**Published:** 2019

**Authors:** Nadia Banihashem, Moghadam Khorasani, Hamidreza Vaffai, Fereshteh Naziri, Soraya Khafri, Shahram Seyfi

**Affiliations:** 1 Clinical Research Development Unit of Ayatollah Rouhani Hospital, Babol University of Medical Sciences, Babol, Iran; 2 Department of Anesthesiology, School of Medicine, Babol University of Medical Sciences, Babol, Iran; 3 Department of Surgery, School of Medicine, Babol University of Medical Sciences, Babol, Iran; 4 Infertility and Health Reproductive Research Center, Health Research Institute, Babol University of Medical Sciences, Babol, Iran

**Keywords:** Coronary arteries bypass grafting, Clopidogrel, Tranexamic acid, Post-operative bleeding, Transfusion

## Abstract

**Background:**

Clopidogrel in combination with aspirin increases bleeding, allogeneic red cell transfusion and reoperation rates after CABG. Tranexamic acid, an antifibrinolytic agent, has been approved for use in cardiac surgery to reduce bleeding. In the present study, we evaluated the impact of tranexamic acid on the transfusion and post-operative blood loss after CABG in patients treated with clopidogrel less than 5 days before surgery.

**Methods:**

This study was a prospective, randomized, double-blinded clinical trial. Patients undergoing on-pump CABG with their last dose of clopidogrel and aspirin less than 5 days preoperatively were randomly assigned to receive tranexamic acid (10 mg/kg before surgical incision and 10 mg/kg after protamine neutralization) or a corresponding volume of saline solution. The incidence of allogeneic red cell transfusion and 48h postoperative blood loss were recorded.

**Results:**

The average volume of blood loss was 776.92±459.81mL for the TXA group and 1075.00±670.91mL for the control group (P=0.03) in the patients with clopidogrel exposure within 48 h before surgery. The average volume of blood loss was not different between two groups in the patients with clopidogrel exposure within 5 days before surgery and also transfusion rate.

**Conclusion:**

The result of this study shows that tranexamic acid reduced blood loss in the patients with clopidogrel exposure within 48 h before surgery. So, it is better that we use tranexamic acid before surgery in all patients.

The main factor of myocardial ischemia is coronary atherosclerosis (1). Clopidogrel is an inhibitor of platelet aggregation that works by irreversible blockade of adenosine diphosphate (2, 3). Clopidogrel has been extensively used in cardiology practice to reduce early stent failure and improve outcomes after acute coronary syndrome (4, 5). In addition, many patients are receiving long-term clopidogrel with or without aspirin for secondary prevention of coronary and cerebrovascular ischemic events. However, some of these patients will go on to require an urgent operative procedure (6). Given these urgent circumstances, it is not always possible for every patient to withhold his or her anticoagulants before surgery (6, 7). Recent studies have shown that clopidogrel treatment in combination with aspirin before CABG is associated with increased postoperative bleeding, transfusion, and re-exploration rates (2-5). The most common cardiac surgery is coronary vessel surgery.

The most complication of post cardiac surgery is bleeding (8). Some drugs used to decrease post cardiac surgery bleeding are aprotinine and aminocaproic acid. Tranexamic acid, a serine protease inhibitor with antifibrinolytic activity, has successfully been used in cardiac and thoracic surgery to reduce overall bleeding and transfusion requirements (9, 10). It is a synthetic anti-fibrinolytic drug that inhibits the lysine-binding site of plasmin and plasminogen and reduces fibrinolysis. Several studies with TXA have demonstrated a significant reduction of perioperative bleeding and the need for transfusions in on-pump as well as off-pump cardiac surgery (9-11). There is only a small number of publications which studied the effects of tranexamic acid on patients undergoing CABG treated with clopidogrel (12, 13). The aim of this study was to determine whether tranexamic acid decreases bleeding and transfusion requirements in patients undergoing CABG treated with clopidogrel less than 5 days before the operation.

## Methods

This prospective double-blind randomized clinical trial study was done on 120 patients (35-70 years) in Ayatollah Rouhani Hospital of Babol University of Medical Sciences undergoing primary isolated on-pump CABG, who received aspirin and clopidogrel with their last ingestion less than 5 days preoperatively in 2016. This research project was approved by the Research Committee of the Babol University of Medical Sciences and by the ethics committee of the same university (ethic code: 3012). The study was registered in the Iranian registration system (IRCT201309125381N8). Written informed consent was provided by all the patients.

 Patients inclusion criteria for the study were those without previous cardiac surgery, creatinine>1.5, hematocrit less than 33%, platelet count less than 50,000/mL, pump time >90 min or blindness of drug. All patients received standard anesthetic induction using a combination of 3 mg/kg of thiopental sodium, 0.1mg/kg of midazolam, 3-4µg/kg of fentanyl and 1–2% of isoflorane. Pancuronium at a dose of 0.1 mg/kg was used to facilitate orotracheal intubation. Intraoperative monitoring included electrocardiogram, pulse oximetry, a radial artery catheter to measure continuous arterial pressure and a central venous pressure line.

Perioperative management of patients was based according to institutional standards by an anesthesiologist and cardiothoracic surgeon who were blinded to the study. Participants were randomly assigned (1:1) to receive either TXA or placebo. An independent statistician was responsible for the computerized sequence generation.

**Figure 1:**
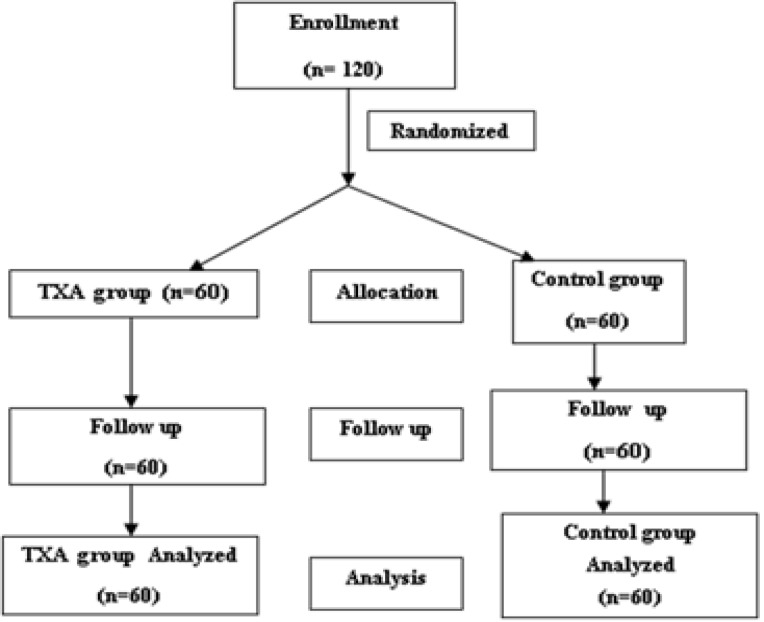
Sampling Diagram of the Study

Patients were consecutively randomly allocated to one of the following two groups: TXA group (n=60) and control group (n=60) (fig 1). Identical syringes of 20 mL labeled with the randomization number contained transparent solution, either tranexamic acid or saline. Patients separated by an anesthesia resident in which these patients received tranexamic acid (10 mg/kg before surgical incision and 10 mg/kg after protamine neutralization) or a corresponding volume of saline solution an expert anesthesilogist.

Surgery was performed through a median sternotomy. The distal ascending aorta was annulated, and a two-stage venous cannula was inserted into the right atrium and inferior vena cava. Anticoagulation for cardiopulmonary bypass was achieved with heparin 400 U/kg through a venous catheter to activated clotting time (Hemochron Jr.) of greater than 480 seconds and monitored during cardiopulmonary bypass. At the completion of cardiopulmonary bypass, heparin was reversed with protamine sulfate (1 mg of protamine for every 100 units of heparin). In the absence of perioperative bleeding, patients received aspirin (80 mg) six h after surgery. Allogeneic packed red blood cells (PRBC) were added in the CPB if hematocrit was <20%. Volume replacement in the ICU was administered using normal saline and lactated Ringer’s solution. PRBC in the ICU was transfused if the hematocrit level was <24%. Fresh frozen plasma was transfused when the postoperative international normalization ratio was >1.5 with excessive bleeding of >200 ml/h for 2 consecutive hours. Platelet concentrates were transfused if the platelet count was <50000 and bleeding occurred (>200 mL/h for 2 h). Surgical re-exploration was indicated when chest tube drainage was >200 ml/h for 6 consecutive hours or >400 ml during the first hour despite normalized activated clotting time and global coagulation status.

The hematocrit level (primary outcome), platelet counts, prothrombin time, and activated partial thromboplastin time were measured 48h after the surgery. The total number of units of packed red blood cells (secondary outcome), fresh frozen plasma, and platelets given during the perioperative period were recorded, including the number of units given during the operation and in the intensive care unit postoperatively. The total blood loss was measured from the chest tube output starting immediately after closure of the chest incision in the operation room and continued until 48 h after surgery. In addition, we assessed the demographic items, length of mechanical ventilation, length of stay in the ICU bedridden and hospital mortality. 

**State statistical: **About 60 patients in each group was determined on the basis of at least decreasing 1.5 unit of blood in TXA group( 10) with power (80%) and a significant level of 0.05 and which assuming 10% possible dropout rate. The data were analyzed using the SPSS for windows (Version 13.0; Spss inc., Chicago, IL, USA). T-test, repeated measurement ANOVA and ANOVA test were used for quantitative factors and chi-square test was used for qualitative factors. In all cases, a p-value of less than 0.05 was considered to be statistically significant.

## Results

The mean duration from clopidogrel administration to CABG was 2.35±0.70 and 2.15±1.70 days for the control and clopidogrel groups, respectively. Aspirin was given until the day before surgery. The elapsed time between the last dose of clopidogrel and the operation was <48 h in 69 of the patients and >48 h in 51 of the patients. Baseline characteristics and risk factors in two groups were presented in table 1. On ICU arrival, the hemoglobin and platelet count significantly decreased in comparison to preoperative values in both groups (table 2).

**Table1:** Baseline Characteristics and risk factors in two groups

	**TXA group**	**Control group**
Age(years)	58.00±9.66	58.30 ± 11.77
Weight (kg)	75.80±14.30	74.50 ± 12.50
Ejection fraction (%)	47.70±8.60	46.00±8.80
Female sex n (%)	15 (25%)	24 (40%)
Hypertension	42(70%)	30(50%)
Diabetes mellitus	36(60%)	18(30%)
History of myocardial infarction	9(15%)	15(25%)
Hyperlipidemia	21(35%)	27(45%)

* Values in table are Mean±SD or n (%)

**Table 2:** Hematocrit in perioperative period in two groups in different times

	**Control group** **(n=60)**	**TXA group** **(n=60)**	**P value ** a
Hematocrit base (%)	37.89±5.20	37.90±4.60	0.99
1 hr postoperative	29.80±3.80	29.90±3.70	0.80
24 hr	30.43±2.10	29.9±3.8	0.38
48 hr	28.03±2.30	27.73±3.50	0.58
P value b	< 0.001	<0.001	

Values in table are Mean± SD

a: The result of the t-test

b : The result of the repeated measurement ANOVA –test

Platelet count was slightly higher in the TXA group (table 3). The average volume of blood loss was 776.92±459.81mL for the TXA group and 1075.00±670.91mL for the control group (P=0.03) in the patients with clopidogrel exposure within 48 h before surgery. The amount of postoperative bleeding and the need for allogeneic transfusions are shown in table 4. There was no significant difference in the volume of postoperative bleeding between the 2 groups at 48 hours after the operation. In this study, 100% of the patients in the two groups received blood products during their hospital stay. The total use of different blood products are depicted in table 5. Perioperative transfusion requirements of PRBC, platelet and FFP did not differ between groups and there were no differences in total doses of heparin (table 5). 

**Table 3:** Platelets in perioperative period in two groups in different time

**Platelet**	**Control group**	**TXA group**	**P-value** a
Base	201000±53884	209300±48150	0.37
1 hr postoperative	137950±37359	157450±39664	0.06
24 h postoperative	152950±39325	163250±46670	0.19
48 h pos-operative	143150±43526	146350±36277	0.66
P value b	<0.001	<0.001	

Values in table are mean± SD

a : The result of the t-test

b : The result of the repeated measurement ANOVA –test

**Table 4:** Comparison of postoperative bleeding in two groups

	**Bleeding** **(first 24h)**	**Bleeding** **(second 24 h)**	**Total bleeding(48h)**
TXA group(n=60)	625.00±405.45	197.50±141.24	822.50±449.50
Control group(n=60)	660.00±405.45	252.50±211.81	912.50±561.22
P-value *	0.62	0.09	0.33

* Values in table are Mean±SD

* The result of the t-test

b: The result of the repeated measurement ANOVA –test

**Table 5:** Perioperative data in two groups

	**Control group**	**TXA group**	**P value** *
Number of grafts	3.05±0.67	3.10±0.89	0.86
Surgery (hours)	5.00 ± 0.60	4.95 ± 0.61	0.18
Cardiopulmonary bypass (minutes)	81.45±27.40	86.95±24.18	0.24
Aortic cross-clamping (minutes)	48.6±20.90	50.25±15.40	0.62
Time to extubation (hours)	8.45±3.92	11.05±14.27	0.18
Length of intensive care unit stay (hours)	78.80±17.98	80.10±28.39	0.78
Transfusion (U)	3.60±1.81	3.20±1.67	0.22
Platelets transfusion	0.8± 1.92	0.85± 1.75	0.66

Values in table are mean± SD

* the result of the t-test

## Discussion

 Several recent studies have demonstrated that clopidogrel within 5 days of CABG is associated with increased blood loss, reoperation rates for bleeding, and increased use of PRBC and platelets (2-6). A study has demonstrated that the reoperation rate in patients undergoing CABG increased from 2.3% to 10.4% when the patients were treated with aspirin or with the combination aspirin and clopidogrel, respectively (14). In a recent study, preoperative clopidogrel exposure increased bleeding and transfusion requirements in patients receiving on-pump CABG. Wade Kang et al. demonstrated that clopidogrel within 3 days preoperatively increases the requirement for blood transfusion in patients undergoing CABG. Waiting more than 3 days decreases blood transfusion requirements. A study indicated that tranexamic acid reduced this risk and provided protection in the patients with clopidogrel exposure within 7 days before surgery. In this study, patients were initially loaded with 10 mg.kg of tranexamic acid followed by a maintenance dose of 10 mg/kg/h until the end of the operation (13). Kang et al. demonstrated that clopidogrel within 3 days preoperatively increased blood loss and transfusion in patients undergoing CABG. Waiting for more than 3 days after the last dose of clopidogrel decreased blood transfusion and bleeding (15). The rate of blood transfusion in the tranexamic acid was less than that in the control group. This is not consistent with our finding. The abovementioned study used 2 g of tranexamic acid while we used 1 g, yet, the results are not much different. The ideal dosage of tranexamic acid remains controversial. 

In our study, the need for blood transfusion was not significantly different in the two groups, although it was less in the tranexamic acid. The possible reason may be the flexibility of blood transfusion criteria and dose of TXA.A study demonstrated that high dose (30 mg/kg bolus followed by 16 mg/kg/h) of TXA is more effective than a low dose (10 mg/kg bolus followed by 1 mg/kg/h) to decrease transfusion needs, blood loss, and repeat surgery (16). 

Weber et al. indicate that tranexamic acid increased arachidonic acid–induced and adenosine diphosphate–induced platelet aggregation in the patients with dual antiplatelet therapy until the day before surgery (9). Indeed, plasmin is a key substance in the effect of tranexamic acid on platelet function. Plasmin activates the complement cascade which impairs platelet function. Plasmin induces proteolytic degradation and redistribution of platelet Ib and IIb/IIIa receptors and thereby reduces platelet adhesion and aggregation. Tranexamic acid reduces plasmin concentration and preventing the binding of plasminogen to fibrin and the conversion of plasminogen to plasmin. Probably, tranexamic acid and preserve platelet function through a reduction of plasmin-induced platelet inhibition (9)..The study contains several limitations. First, it was done just in a one center study with a relatively small sample size and short follow-up period. Second, this study did not examine the impact of dose of clopidogrel on bleeding outcome in patients. In conclusion, TXA decreases bleeding after CABG in patients treated with clopidogrel<48 h before surgery. Further work is required to determine the optimal strategy in patients who require CABG without clopidogrel and aspirin cessation. 
